# Laparoscopic radical gastrectomy for gastric cancer following liver transplantation: a report of a rare and complex case and a multidisciplinary management strategy

**DOI:** 10.3389/fmed.2026.1914424

**Published:** 2026-08-13

**Authors:** Ying Miao, Enhong Zhao, Shuai Zheng, Ji Yang, Ziyu Yue

**Affiliations:** Affiliated Hospital of Chengde Medical University, Chengde, China

**Keywords:** gastrectomy, laparoscopic surgery, liver transplantation, metabolic syndrome, transplant immunology

## Abstract

*De novo* gastric cancer after liver transplantation is clinically rare, and radical resection remains the only potentially curative treatment. However, post-transplant anatomical distortion and extensive intra-abdominal adhesions increase surgical complexity, while long-term immunosuppression elevates the risks of perioperative infection and organ dysfunction. Furthermore, chemotherapy and immune checkpoint inhibitors may precipitate graft rejection. We report a 68-year-old man who underwent liver transplantation 18 years earlier for HBV-related hepatocellular carcinoma (T1aN0M0), meeting the Milan criteria. Preoperatively, serum HBV DNA was 2.3 × 10^3^ IU/mL. He received entecavir and basiliximab induction, followed by early triple immunosuppression and long-term tacrolimus maintenance. During follow-up, HBV DNA remained undetectable, liver function was stable, and no transplant-related complications occurred. Eighteen years after transplantation, he presented with upper abdominal pain. Imaging and endoscopy demonstrated cT4N1M0 gastric adenocarcinoma of the antrum with hepatic parenchymal invasion. Following multidisciplinary discussion, laparoscopic D2 radical gastrectomy with Roux-en-Y reconstruction, partial hepatectomy, and ultra-tension-reducing abdominal wall closure were performed. The postoperative course was uneventful. This case suggests that multidisciplinary, standardized perioperative management can enable safe radical laparoscopic gastrectomy in selected liver transplant recipients.

## Introduction

Malignancies develop in 1–16.5% of solid-organ transplant recipients, predominantly as skin cancers and post-transplant lymphoproliferative disorders (1). *De novo* gastric cancer after liver transplantation is rare, and radical gastrectomy remains the standard curative treatment (2). However, surgery is complicated (3) by dense intra-abdominal adhesions and distorted hilar anatomy, increasing the risks of vascular or biliary injury and hemorrhage. Chronic immunosuppression impairs immune surveillance and increases susceptibility to infection, whereas chemotherapy or immunotherapy may trigger graft rejection or liver injury. We report a case of locally advanced gastric cancer after liver transplantation, highlighting multidisciplinary management, minimally invasive surgery, immunosuppressive adjustment, and postoperative surveillance.

## Case report

### Medical history

A 68-year-old man who underwent a liver transplant 18 years ago following HBV-related hepatocellular carcinoma. He has been on long-term maintenance therapy with tacrolimus and entecavir since the operation. At the same time, he underwent a TIPS procedure for upper gastrointestinal haemorrhage. He was admitted to hospital on 7 April 2026 with persistent upper abdominal pain and was diagnosed with a malignant tumour of the stomach.

### Preoperative examination

**Figure 1:**
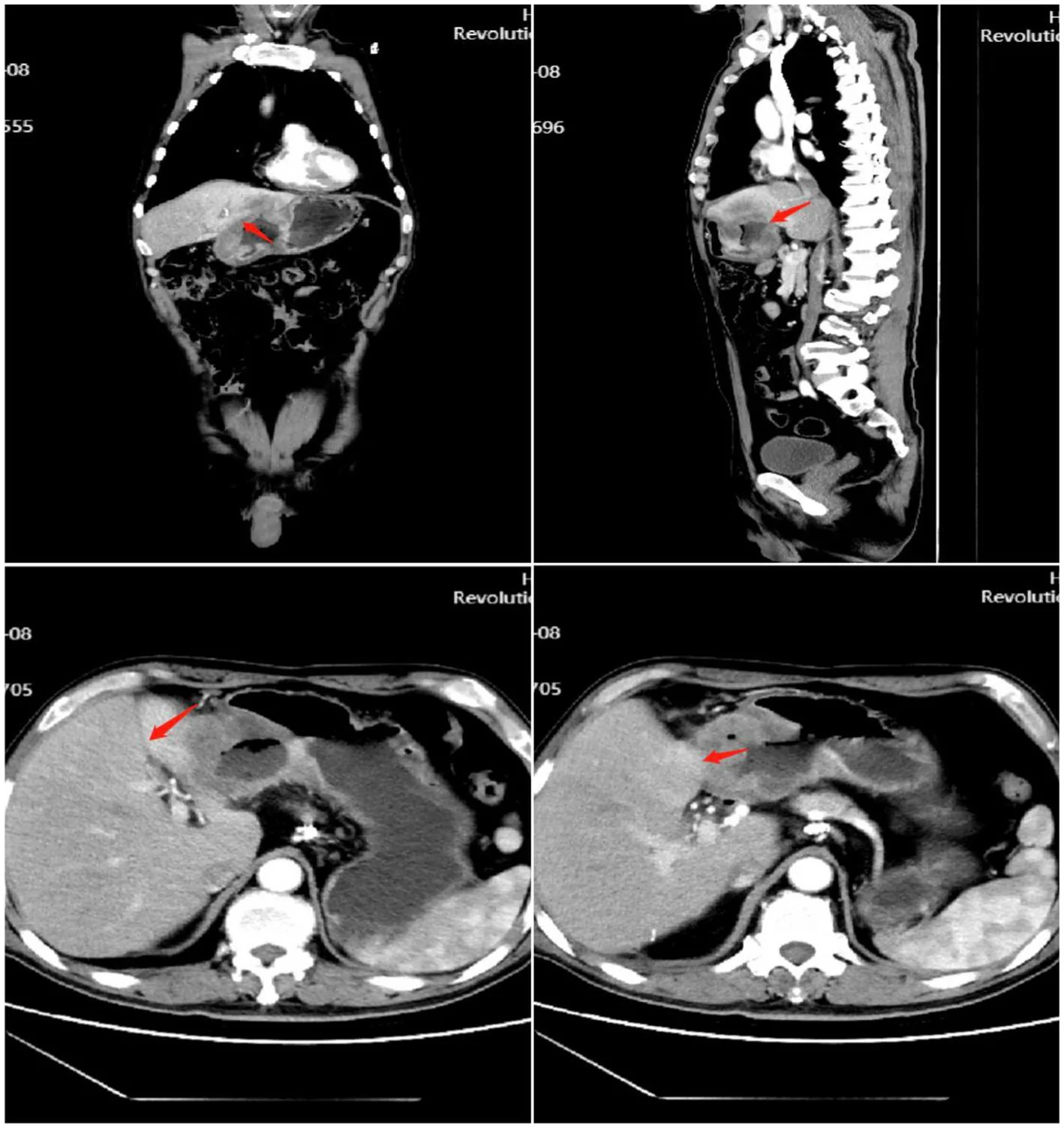
This is a high-resolution CT scan of the abdomen, showing coronal, sagittal, and transverse views; the red arrows indicate the gastric cancer invading the transplanted liver.

Gastroscopy and histopathological examination confirmed invasive adenocarcinoma of the gastric antrum, with the lesion compressing the pylorus; abdominal CT scan revealed tumour invasion of the liver and enlarged perigastric lymph nodes, accompanied by post-liver transplant and post-cholecystectomy changes. Gastrointestinal tumour markers CEA, CA19-9, CA72-4 and CA242 were significantly elevated; chest and cranial CT scans ruled out distant metastases. The patient’s cardiac, pulmonary, hepatic and renal functions are normal, and there are no contraindications to surgery; however, urgent surgical intervention is required due to pyloric obstruction Figure 1.

### Multidisciplinary team (MDT) assessment before surgery

A multidisciplinary team of specialists jointly assessed the surgical risks: extensive intra-abdominal adhesions following liver transplantation obscured the anatomy of the hepatic hilum, and the tumour involvement of the liver necessitated a partial hepatectomy, significantly increasing the risks of intraoperative haemorrhage, vascular injury and postoperative liver dysfunction; discontinuing tacrolimus for 3–5 days prior to surgery posed a risk of graft rejection, requiring dynamic monitoring of liver function during the perioperative period and the earliest possible resumption of immunosuppression; Prolonged immunosuppression increases the risk of postoperative infection, haemorrhage, respiratory failure and multi-organ failure; the patient will therefore be transferred to the ICU for close monitoring following surgery. Following a comprehensive assessment by a multidisciplinary team of specialists, it was decided to proceed with laparoscopic D2 radical gastrectomy for gastric cancer with Roux-en-Y anastomosis, combined with partial hepatectomy and tension-relieving suturing of the abdominal incision.

### Perioperative management

Before radical gastrectomy, a multidisciplinary team developed an individualized perioperative management protocol. The patient and family received detailed counseling regarding the heightened risks of gastric cancer surgery after liver transplantation, including graft rejection during tacrolimus interruption, opportunistic infections associated with chronic immunosuppression, and concomitant gastrointestinal and hepatic dysfunction. The rationale for balanced immunosuppression, scheduled laboratory surveillance, and emergency response pathways was explained, and written informed consent was obtained.

Tacrolimus was withheld for 3–5 days before surgery to reduce bleeding and infectious risks. During withdrawal, liver biochemistry (ALT, AST, and total bilirubin) and coagulation parameters were assessed daily to detect early rejection. Postoperatively, tacrolimus was resumed at the maintenance dose (0.1–0.2 mg/kg twice daily) via a jejunal feeding tube. Trough concentrations were measured every 2–3 days, targeting 3–5 ng/mL.

Contingency plans addressed rejection and severe infection. Suspected rejection, indicated by elevated liver enzyme levels, prompted intravenous methylprednisolone pulse therapy; persistent biochemical abnormalities unresponsive to corticosteroids warranted percutaneous liver biopsy. Broad-spectrum antimicrobial prophylaxis was administered perioperatively, with serial blood cultures, chest CT, CRP, and procalcitonin monitoring. Confirmed severe bacterial or fungal infection prompted tacrolimus dose reduction and intensified pathogen-directed therapy.

### Intraoperative findings and procedures

Intraoperative exploration revealed extensive adhesions within the abdominal cavity. A 5 × 6 cm tumour in the body of the stomach had penetrated the serosa and involved the liver and the capsule of the pancreas; 3–8 groups of perigastric lymph nodes were enlarged and fused, with the lymph nodes in group 8 encircling the major blood vessels. During the operation, three-quarters of the distal stomach, part of the liver and the surrounding affected tissue were resected; the proximal stomach was preserved and a Roux-en-Y anastomosis was performed, with the anastomotic site reinforced. The hepatic hilum was meticulously dissected, and the affected liver tissue was completely resected to achieve negative surgical margins; intraoperative blood loss was minimal.

## Postoperative management and clinical course

Cefmetazole was administered postoperatively to prevent infection, and inflammatory markers were monitored closely; the medication was discontinued on the fifth day once the markers had returned to normal (see the below table for specific values).

**Table 1:** 

Detection time	White blood cells WBC (×10^9^/L)	Neutrophil percentage EN%	CRP (mg/L)	PCT (ng/ml)
Postoperative day 1	11.0 × 10^9^/L	78.60%	64 mg/L	0.842 ng/mL
Postoperative day 3	8.9 × 10^9^/L	65.10%	79.04 mg/L	0.308 ng/mL
Postoperative day 5	6.66 × 10^9^/L	58.10%	9.20 mg/L	0.056 ng/mL
Postoperative day 11 (prior to discharge)	5.3 × 10^9^/L	48.9%	1.1 mg/L	0.03 ng/mL

Six hours post-operatively, tacrolimus was administered via the jejunal feeding tube at the original maintenance dose (0.1–0.2 mg/kg, twice daily). Trough plasma concentrations and liver function were monitored daily; plasma concentrations were maintained within the therapeutic range throughout, and there were no instances of transplant rejection or intra-abdominal haemorrhage during the entire course of treatment. The patient resumed oral intake on the ninth post-operative day; there was no anastomotic fistula, the surgical incision healed well, and the patient was discharged without complications.

**Figure 2:**
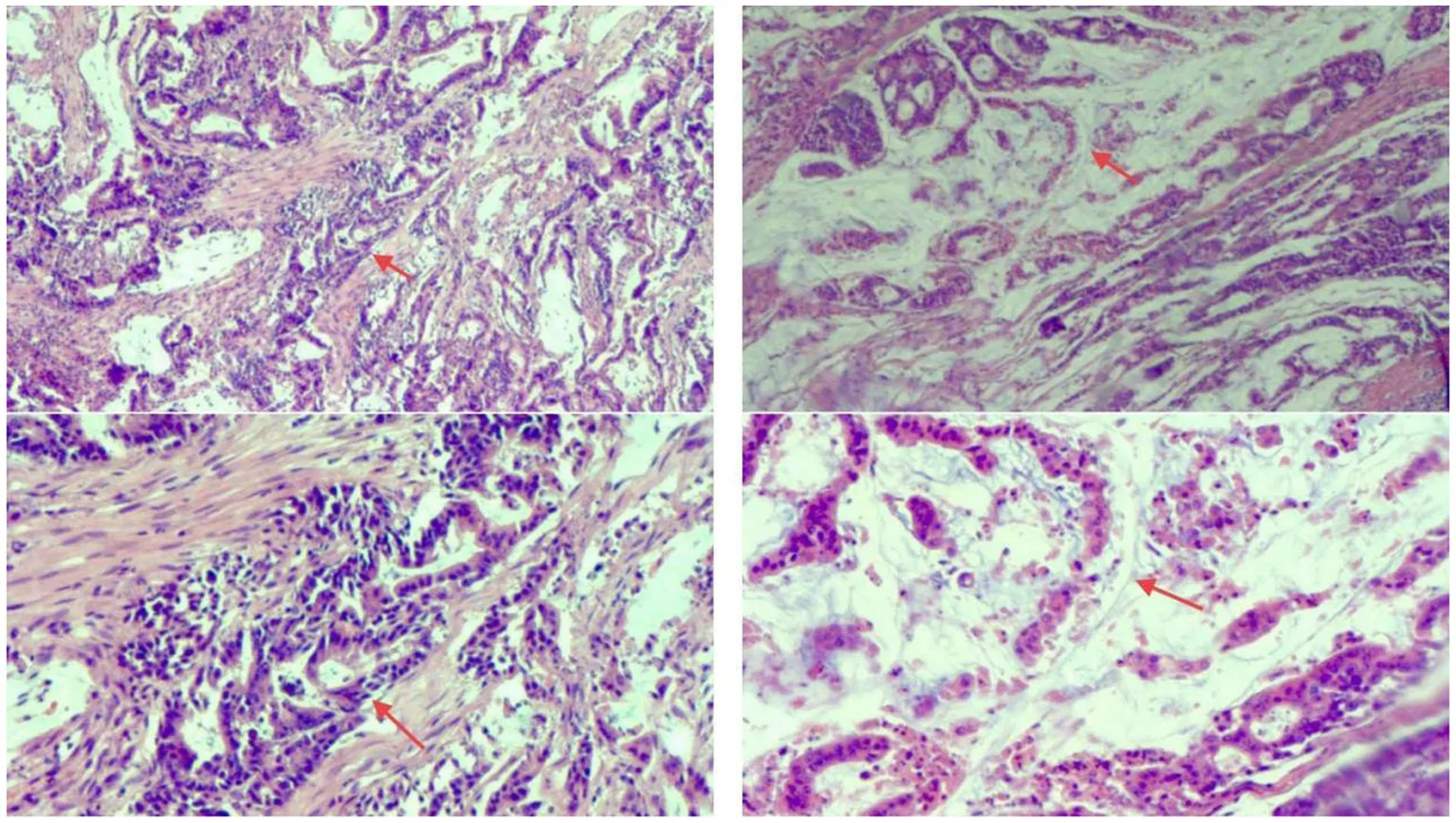
On the left is a moderately to poorly differentiated adenocarcinoma (top: ×40; bottom: ×100); on the right is a mucinous adenocarcinoma (top: ×40; bottom: ×100).

**Figure 3:**
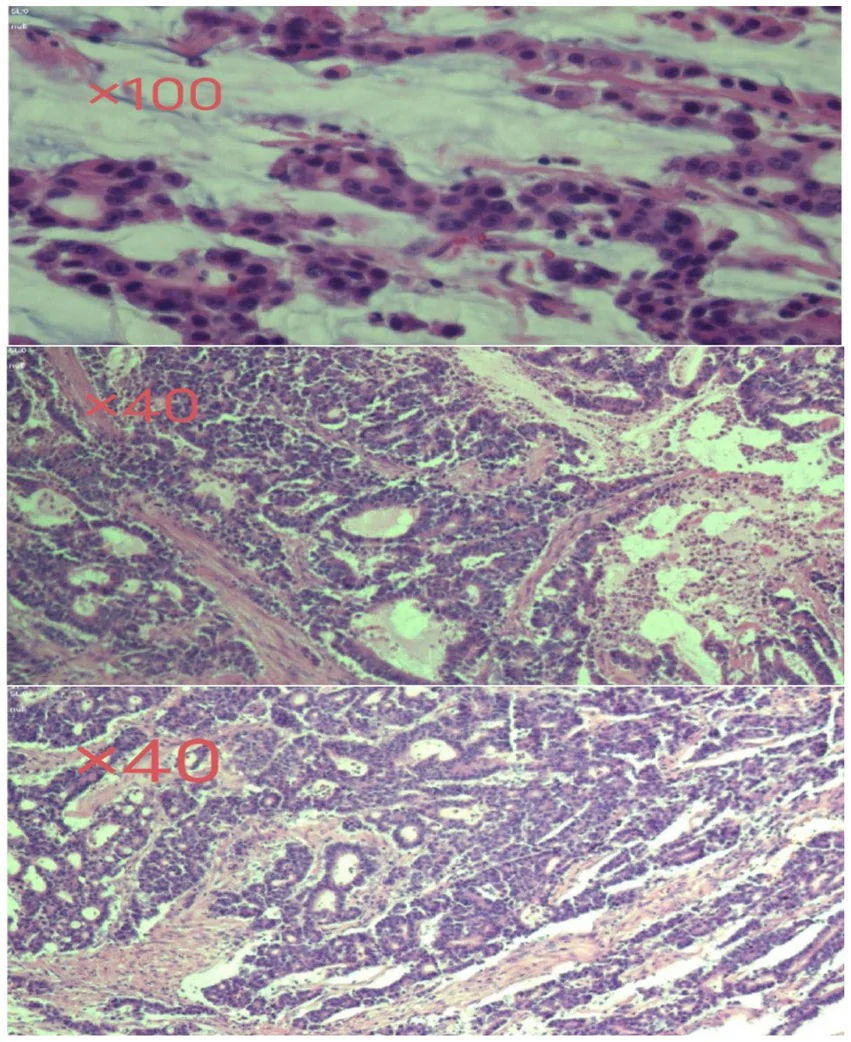
Cerative moderately to poorly differentiated adenocarcinoma with features of mucinous adenocarcinoma.

Pathological examination revealed a moderately to poorly differentiated mucinous adenocarcinoma of the stomach, invading the full thickness of the wall and involving nerves; there was suspicious involvement of liver tissue. No metastases were detected at the surgical margins or in the sent lymph nodes. Immunohistochemistry demonstrated a lack of mismatch repair proteins, low HER-2 expression, a Ki-67 index of 70 per cent, and negative results for viral and BRAF mutations. A consultation with the Oncology Department is planned 2 weeks after discharge to formulate an adjuvant treatment plan Figures 2, 3.

## Ethics and consent

This case report has been submitted to the hospital’s Ethics Committee, and written informed consent has been obtained from the patient, authorising the publication of all clinical data and imaging findings. Written informed consent to the publication of this case report has been obtained from the patients.

## Discussion

Post-transplant *de novo* gastric cancer (GC) is relatively rare in clinical practice. International survey studies indicate that the incidence of *de novo* gastric malignant tumors after transplantation is approximately 0.445%, while the occurrence rate of malignant tumors in liver transplant recipients is about twice that of the general population (4). Although the proportion of newly developed gastrointestinal malignant tumors is low, their occurrence can significantly reduce survival rates. Research shows that *de novo* malignant tumors have become the second leading cause of death among organ transplant recipients (5). Under long-term immunosuppressive conditions, the clinical progression of gastric cancer may be more rapid and aggressive, making *de novo* malignant tumors one of the important causes of death in patients after organ transplantation (6). Therefore, it is of great significance to develop reasonable treatment plans based on individual patient circumstances and to achieve early detection of *de novo* gastric cancer through long-term, close follow-up in order to improve patient prognosis and prolong long-term survival.

The occurrence mechanism of newly developed gastric cancer after liver transplantation may be related to the combined effects of various factors such as long-term immunosuppression, opportunistic infections, and metabolic syndrome. Firstly, Transplant recipients require long-term medication to prevent rejection; as a result, their immune surveillance function is compromised, leading to an increased risk of tumour development. First-line calcineurin inhibitors (CNIs, such as tacrolimus and cyclosporine) not only suppress the immune system but may also enhance tumour invasiveness and metastatic potential by impairing DNA repair, upregulating VEGF and activating the TGF-β1 pathway (7). Secondly, post-transplant opportunistic infections may also play a role in the development and progression of gastric cancer following liver transplantation. Systemic immunosuppression renders liver transplant recipients more susceptible to new bacterial, viral and fungal infections, and may also lead to the reactivation of previously latent infections (8). In addition, the application of immunosuppressants after liver transplantation may increase the risk of *Helicobacter pylori* (Hp) infection (9). Therefore, opportunistic infections after transplantation may promote the occurrence of gastric cancer through mechanisms such as chronic inflammation, immune regulation abnormalities, and activation of pro-carcinogenic signaling pathways. Strengthening infection screening, monitoring, and intervention after transplantation may help reduce the risk of related tumors.

Metabolic syndrome following liver transplantation is also a potential risk factor for the development of *De novo* gastric cancer. Although liver transplantation improves survival in patients with end-stage liver disease, metabolic syndrome continues to impair the function of the transplanted liver and affects long-term prognosis; clinically, it is characterised by obesity, dyslipidaemia, hypertension and insulin resistance. Immunosuppressants such as glucocorticoids, CNIs and sirolimus may increase the risk of developing diabetes: these drugs promote glucose release, inhibit insulin secretion and reduce insulin sensitivity; at the same time, they cause elevated blood lipid levels by regulating hepatic lipid synthesis and lipoprotein metabolic pathways. CNI drugs may also constrict renal blood vessels, leading to water and sodium retention and inducing post-operative hypertension (10). Metabolic syndrome constitutes a state of chronic low-grade inflammation, which can create a tumour-promoting microenvironment and increase the risk of gastric cancer (11). Therefore, implementing integrated management of body weight, blood glucose, blood lipids and blood pressure in transplant patients can reduce the risk of gastric cancer and other malignant tumours.

**Figure 4:**
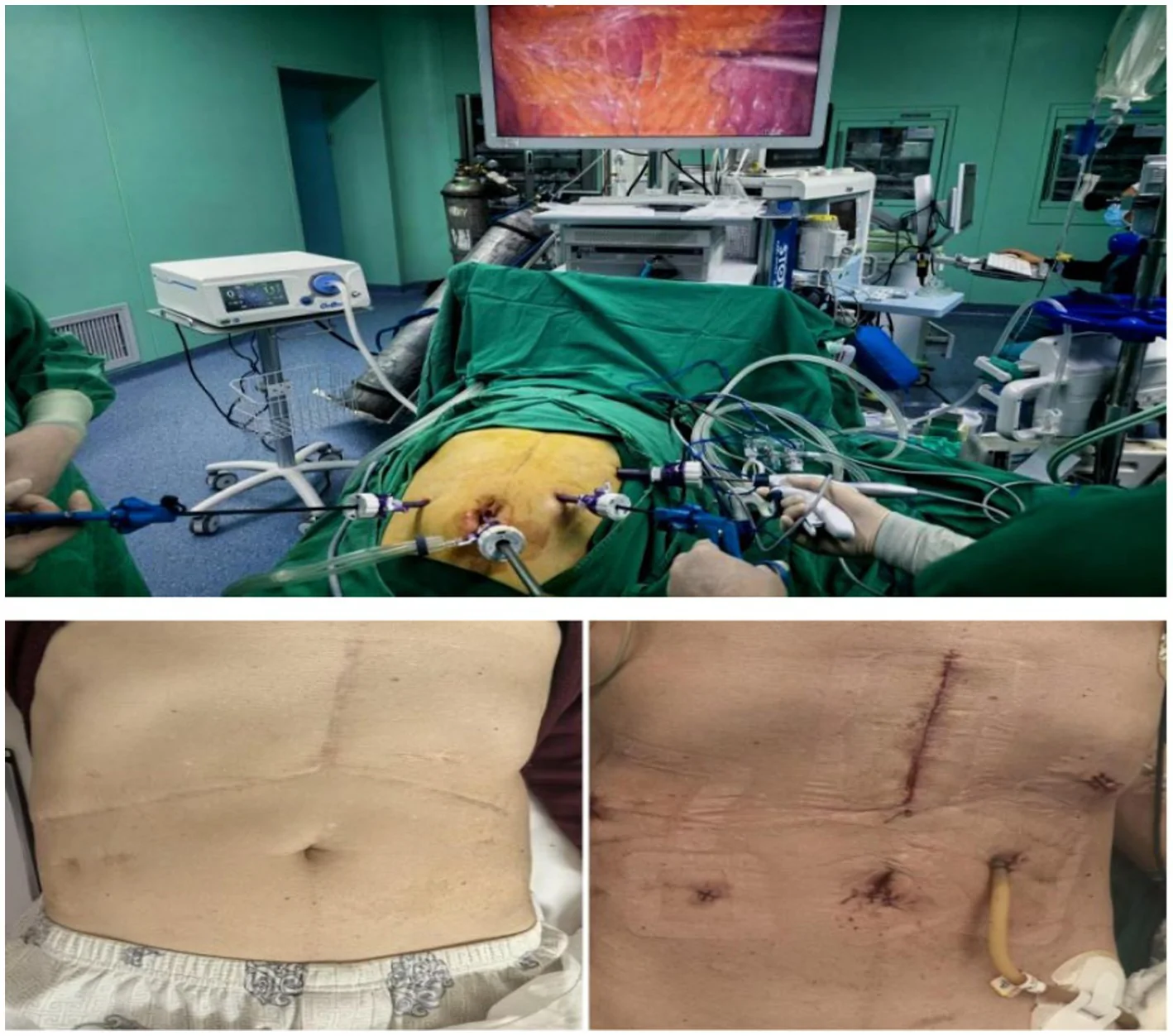
Minimal trauma and panoramic visual field of laparoscopic gastrectomy.

Compared with primary gastric cancer, gastric cancer surgery after liver transplantation is more challenging. Post-transplant extensive abdominal adhesions and remodelled hepatic portal vessels disrupt lymphatic drainage of No.5, 8 and 12 lymph nodes, raising difficulties in dissection and vascular handling, so open surgery was conventionally adopted (2). As laparoscopic techniques mature, minimally invasive radical gastrectomy shows prominent value for transplant recipients. Laparoscopy features minimal trauma, mild postoperative pain, fast intestinal recovery and shorter hospitalization. Its magnified vision enables precise operation and less blood loss, lowers postoperative ileus by reducing intestinal traction, relieves surgical stress and ensures satisfactory safety (12), bringing superior postoperative recovery for liver transplant patients Figure 4.

Adjuvant therapy following radical gastrectomy for gastric cancer in liver transplant recipients presents numerous challenges. Transplant patients generally have a poorer overall tumour prognosis, and anti-tumour regimens are constrained by multiple factors, including graft toxicity, the risk of rejection, liver and kidney damage, and underlying comorbidities (13). Patients with gastric cancer who have undergone D2 radical resection can derive significant benefit from adjuvant chemotherapy (5). The first-line chemotherapeutic agent capecitabine has been shown in animal models to suppress acute graft rejection (14); rhythm-alternating chemotherapy modulates the Nrf2/HO-1/GPX4 pathway, induces ferroptosis in T cells, and alleviates liver transplant rejection (15). Results of this preclinical animal study cannot be completely extrapolated to human liver transplant patients. The clinical implementation of adjuvant chemotherapy requires a balance to be struck between the benefits of tumour treatment and the risks of graft damage, myelosuppression, and hepatic and renal toxicity. Immune checkpoint inhibitors (ICIs) have demonstrated definite efficacy against various advanced tumours; however, PD-1/PD-L1 are co-expressed on both transplanted tissues and alloreactive T cells (16), meaning that their use may easily activate rejection-inducing T cells, leading to graft rejection or even graft failure. Therefore, prior to the use of ICIs in liver transplant patients, a comprehensive assessment of tumour stage, graft function, risk of rejection and general health status is required, with an individualised treatment plan developed by a multidisciplinary team.

In the context of post-liver transplant immunomodulation and the comprehensive treatment of gastric cancer, the mTOR inhibitor rapamycin exerts a dual action. Traditional immunosuppressive regimens, primarily based on calcineurin inhibitors (CNIs), can effectively prevent rejection but increase the risk of *de novo* tumours. Rapamycin possesses both immunosuppressive and antitumour activity; by regulating multiple pathways, it downregulates VEGF, induces tumour cell apoptosis, and inhibits tumour proliferation and progression (6); at the same time, it optimises the T-cell response, enhances the control of cytomegalovirus (CMV) infection and reduces opportunistic infections (17). For patients who have undergone liver transplantation and have concurrent gastric cancer, switching to an immunosuppressive regimen centred on mTOR inhibitors can simultaneously protect the graft, reduce the risk of rejection and infection, and exert an antitumour effect; however, its individualised efficacy and safety still require validation through further prospective studies.

*De novo* gastric cancer after liver transplantation is caused by a combination of immunosuppression, infection, metabolic disorders and conventional risk factors; therefore, enhanced early screening and diagnosis post-surgery are required. Laparoscopic radical resection shows promising prospects when performed following rigorous assessment; subsequent comprehensive treatment must strike a balance between tumour control, anti-rejection therapy and organ safety. Dual-action mTOR inhibitors represent a potential new therapeutic direction, although large-scale, multicentre studies are still required to clarify the underlying mechanisms and develop personalised treatment regimens.

## Data Availability

The raw data supporting the conclusions of this article will be made available by the authors, without undue reservation.
